# FOLFOXIRI Resistance Induction and Characterization in Human Colorectal Cancer Cells

**DOI:** 10.3390/cancers14194812

**Published:** 2022-09-30

**Authors:** George M. Ramzy, Laura Boschung, Thibaud Koessler, Céline Delucinge-Vivier, Mylène Docquier, Thomas A. McKee, Laura Rubbia-Brandt, Patrycja Nowak-Sliwinska

**Affiliations:** 1 Molecular Pharmacology Group, School of Pharmaceutical Sciences, Institute of Pharmaceutical Sciences of Western Switzerland, University of Geneva, 1211 Geneva, Switzerland; 2 Institute of Pharmaceutical Sciences of Western Switzerland, University of Geneva, 1211 Geneva, Switzerland; 3 Translational Research Center in Oncohaematology, 1211 Geneva, Switzerland; 4 Department of Oncology, Geneva University Hospitals, 1205 Geneva, Switzerland; 5 iGE3 Genomics Platform, University of Geneva, 1211 Geneva, Switzerland; 6 Department of Genetics & Evolution, University of Geneva, 1211 Geneva, Switzerland; 7 Division of Clinical Pathology, Diagnostic Department, University Hospitals of Geneva (HUG), 1211 Geneva, Switzerland

**Keywords:** FOLFOXIRI, drug-resistance, colorectal cancer

## Abstract

**Simple Summary:**

We created colorectal cancer in vitro models to study how an induced drug resistance profile can alter cell response and sensitivity to a treatment. By chronically exposing the cells to current first-line treatments (5-FU+folinic acid+oxaliplatin+SN38), resistance to the chemotherapy was obtained. We further investigated the mechanism underlying the acquired chemoresistance and highlighted the main up- and downregulated genes implicated. We also showed that optimized drug combination composed of tyrosine kinase inhibitors overcome chemotherapy-induced resistance.

**Abstract:**

FOLFOXIRI, i.e., the combination of folinic acid, 5-fluorouracil, oxaliplatin, and irinotecan, is a first-line treatment for colorectal carcinoma (CRC), yet non-personalized and aggressive. In this study, to mimic the clinical situation of patients diagnosed with advanced CRC and exposed to a chronic treatment with FOLFOXIRI, we have generated the CRC cell clones chronically treated with FOLFOXIRI. A significant loss in sensitivity to FOLFOXIRI was obtained in all four cell lines, compared to their treatment-naïve calls, as shown in 2D cultures and heterotypic 3D co-cultures. Acquired drug resistance induction was observed through morphometric changes in terms of the organization of the actin filament. Bulk RNA sequencing revealed important upregulation of glucose transporter family 5 (GLUT5) in SW620 resistant cell line, while in the LS174T-resistant cell line, a significant downregulation of protein tyrosine phosphatase receptor S (PTPRS) and oxoglutarate dehydrogenase-like gene (OGDHL). This acquired resistance to FOLFOXIRI was overcome with optimized low-dose synergistic drug combinations (ODCs) acting via the Ras-Raf-MEK-ERK pathway. The ODCs inhibited the cell metabolic activity in SW620 and LS174T 3Dcc, respectively by up to 82%.

## 1. Introduction

Colorectal cancer (CRC) is the third most diagnosed and second deadliest cancer worldwide, with a reported incidence of 1.9 million cases in 2020 [1]. It results from a complex disease process implicating both genetic factors and environmental exposure, including the inflammatory conditions of the digestive tract. In primary tumors, patients undergo surgery with chemotherapy determined by the stage of the disease and the patient’s condition. However, only few patients with metastatic CRC undergo surgery with curative results. The first-line treatment option for CRC patients diagnosed with advanced disease is the combination of chemotherapeutics defined by clinical practice guidelines [2,3]. The choice of medication is based on estimations of the goals of the therapy, the mutational profile of the tumor, and the toxicity profiles of the composing drugs. These typically include fluorouracil (5-FU) and folinic acid (FA). 5-FU is an antimetabolite that inhibits the incorporation of different nucleotides into RNA and DNA, as well as a direct inhibitor of the enzyme thymidylate synthase [4]. FA potentiates the cytotoxic effects of 5-FU through the inhibition of fluoronucleotide synthesis by competing with the natural substrates of thymidylate synthase. These two drugs have been considered the backbone of chemotherapeutic modalities for CRC patients by efficiently inhibiting tumor progression, and extending the median overall survival of late-stage CRC patients [5]. Oxaliplatin, a platinum-based compound, forms different types of adducts with the DNA strands resulting in irreversible transcriptional errors, leading to caspase 3 activation and apoptosis induction [6,7]. Oxaliplatin is widely administered with 5-FU/FA (FOLFOX), and studies have shown an increase in the median overall survival by up to 12 months [8]. The addition of irinotecan and its active metabolite SN38, a topoisomerase I inhibitor, to the standard doublet (FOLFIRI) or triplet (FOLFOXIRI) chemotherapy, increased the median overall survival of metastatic CRC patients. However, this improvement has been detrimental to the overall quality of life due to increased toxicity and side effects [9].

The major issue with this standardized treatment protocol is that it fails to identify the cohort of CRC patients with poor prognosis that should receive adjuvant therapy and especially fails to predict which patients will benefit from a given treatment. Focus has been brought on improving the risk/benefit ratio by extending the treatment repertoire, in the metastatic setting, to include biologically targeted agents. This includes epidermal growth factor receptor (EGFR) inhibitors, such as cetuximab or panitumumab [10], and anti-angiogenic agents targeting vascular endothelial growth factor (VEGF) such as bevacizumab [11], in addition to the broad spectrum small-molecule-based tyrosine kinase inhibitor regorafenib. This resulted in an improved median overall survival of patients with metastatic CRC [12]. In spite of advances in treatment modalities over the past decade, the 5-year survival rate is still poor, and the main reason for treatment failure is the development of acquired resistance to all standard therapy, which appears in 90% of patients with metastatic cancer [13].

Malignant tumors can have intrinsic resistance to chemotherapy, which is important in defining the initial and subsequent treatment modalities. For instance, the elucidation of the presence of *RAS* mutations in CRC highlighted the innate resistance to anti-EGFR therapeutic options allowing the clinical adjustment [14]. On the other hand, acquired resistance to one drug may confer resistance to other drugs [15]. Multiple resistance mechanisms exist for all cytotoxic therapies (chemoresistance) and each targeted pathway. For traditional chemotherapy, resistance relates to decreased drug delivery to or drug uptake by the cancer cell, or by an enzymatic conformational change that affects the metabolism of the drug [16]. Resistance to targeted therapies resides in mutation, up/downregulation and activation of molecules downstream of a specific signaling pathway, or in some cases in a pathway bypass mechanism [17,18]. Studies have linked multi-drug resistance (MDR) in cancer cells to the metabolism of lipids. The modulation in the phospholipid composition of the plasma membrane was shown to affect membrane fluidity as well as the drug binding affinity through structural changes in different micro-domains of the cell membrane [19].

The dense desmoplastic mass, which is the result of severe remodeling of the connective tissue, along with cancer-associated fibroblasts (CAFs), immunosuppressive tumor microenvironment (TME), and extracellular matrix stiffening, is known as a main player in chemoresistance in colorectal cancer treatment [20]. These led to the development of immunotherapeutic approaches, as well as anti-stromal treatments, which to date have largely failed. Thus, there is still an unmet need to better understand the underlying mechanism of CRC chemoresistance, in order to open an alley to more promising clinical results.

The important outstanding questions include how the chronically treated cancer cells with the above-mentioned chemotherapeutic combinations differ from treatment-naïve cells, and how would they respond to targeted drugs. To address these questions, we have previously established optimized drug combinations of FOLFOXIRI, specific to four human CRC cell lines characterized by different origins and mutational status [21]. There are publications on the establishment of resistance to single drugs, i.e., 5-FU [22]. OX [23], SN38 [24], but induction of resistance to FOLFOXIRI has not been reported.

In this study, we generated FOLFOXIRI-resistant cells by chronically treating them with cell-line-specific doses of FOLFOXIRI [21]. We compared their cell morphology and transcriptome versus treatment-naïve parental cells. We further exposed them to optimized multidrug mixtures of tyrosine kinase inhibitors in 2D and 3D co-cultures and compared their sensitivity to those treatments.

## 2. Materials and Methods

### 2.1. Cells and Cell Culture Conditions

Human CRC cells HCT116, LS174T, DLD1 and SW620, see Table 1,were purchased at ATCC or Public Health England, while the human immortalized endothelial cells ECRF24 [25] were generated at the Vrije Universiteit Amsterdam, The Netherlands. DMEM Glutamax medium (31966-021, Gibco, Gaithersburg, MD, USA) was used in the culture of HCT116, LS174T, and SW620 cells., RPMI-1640 Glutamax medium (1870-010, Gibco) for DLD1 cells, EMEM medium (M2279, Sigma-Aldrich, Buchs, Switzerland) supplemented with 2mM L-Glutamin (25030024, Gibco) for CCD18co cells and a mixture of DMEM/RPMI 1:1 for ECRF24 cells. The latter were seeded on 0.2% gelatin-coated surface (G1393, Sigma-Aldrich). All culture media were supplemented with 10% fetal bovine serum (S1810-500, Biowest, Nuaillé, France) and 1% penicillin/streptomycin (4-01F00-H, Bioconcept, Basel, Switzerland). Cells were kept in a humidified atmosphere at 37 °C with 5% CO_2_ (Binder). All cells were tested for mycoplasma presence before all experiments. All cells were seeded in 96-well plates (353072, Corning, NY, USA) at the following densities; HCT and DLD1 at 2500 cells/well, LS174T at 3500 cells/well and SW620 at 5000 cells/well, respectively.

### 2.2. Heterotypic 3D Co-Cultures

CRC cells were seeded in a clinically relevant 1:1 ratio with CCD18co fibroblasts and 5% ECRF24 endothelial cells in 96-well U-bottom low attachment plates (650970, Greiner Bio-One, Frickenhausen, Germany) [31,32]. The 3Dcc culture media contained a mixture of DMEM-RPMI-EMEM (1:1:1) supplemented with 2.5% Matrigel^®^ (354254, Corning, Bedford, MA, USA). The 3D-CCs were treated with drugs 48 h post-seeding.

### 2.3. FOLFOXIRI Resistance Induction

LS174T, HCT116, DLD1 and SW620 cells were chronically exposed to their corresponding FOLFOXIRI mixture, see Table 2, once weekly in their culture flask (T75) for at least 34 weeks. The FOLFOXIRI treatment was kept for 72 h, then cells were washed twice with PBS that was subsequently replaced with corresponding fresh medium till the next treatment point. Every two weeks, a cell metabolic activity assay (CellTiter-Glo^®^, Promega, Madison, WI, USA) was performed to evaluate the decrease in cell sensitivity to the chemotherapy chronic treatment and compared to the treatment-naïve non-treated cells.

### 2.4. Drugs

All drugs were aliquoted, stored at −80 °C and thawed prior to each experiment for one-time use. Regorafenib (R-8024), erlotinib (E-4007), vemurafenib (V-2800) were obtained from LC laboratories (Woburn, MA, USA) and diluted in sterile DMSO, respectively, to a concentration of 20 mg/mL, 15 mg/mL, 20 mg/mL; Selumetinib (HY-50706) and GDC-0994 (HY-15947), SN38 (29112) were obtained from MedChemExpress (Monmouth Junction, NJ, USA) and diluted, respectively, in sterile DMSO to a concentration of 20 mg/mL, 10 mg/mL, and 1mg/mL. 5-fluorouracil (F6627, Sigma-Aldrich) and folinic acid (F787, Sigma-Aldrich) were dissolved, respectively, in sterile DMSO at 10 mg/mL and 20 mg/mL. oxaliplatin (O9512, Sigma-Aldrich) in UltraPure distilled sterile water at a concentration of 5 mg/mL. 2D cell cultures were exposed to the different drug mixtures for 72 h or 72 h + 72 h at 24 h post-seeding, while 3Dcc were exposed to treatment after 48 h post-seeding for only 72 h. Cell culture media with and without 0.15% DMSO were used as controls.

### 2.5. Drug-Doses Conversion

The calculation of the clinically used dose (CUD) was based on values obtained from published pharmacokinetic studies performed in patients exposed to each drug at standard or maximum tolerated doses. The area under the curve (AUC_0–24h_), which corresponds to the plasma concentration of the drug over the first 24 h, was set as a basis of the average drug concentration. The CUDs were 0.49 µM, 9.61 µM, 0.39–0.59 µM and 0.1 µM for folinic acid [33], 5-fluorouracil [34], oxaliplatin [35,36] and irinotecan/SN-38, respectively [35,37].

### 2.6. Cell metabolic Activity Assay

Cell viability was evaluated through metabolic activity using CellTiter-Glo^®^ bioluminescence-based assay (G7572, Promega). The measurements were performed using the BioTek Cytation 3 with Gen5 Image software (version 3.04) [21,38].

### 2.7. Immunofluorescence Staining

Immunofluorescence staining was performed on CRC-FX-R and CRC treatment-naïve cells cultured in 24-well plates using cytoskeleton (F-actin) and nuclear (DAPI) staining. First, the cells were fixed using a 2% formaldehyde solution for 10 min, then washed twice with PBS, followed by a permeabilization step using Triton-X at a concentration of 0.1% in PBS for 15min. Then, the cells were exposed to a 1% BSA solution to block unspecific binding sites for 20min. Cells were then stained for F-actin with Phalloidin Flash-488 diluted at a 1:200 ratio (424201, Biolegend, San Diego, CA, USA) for 20 min at room temperature, followed by a double washing step using PBS prior to DAPI staining (diluted at 1:5000) for 5 min. Cells were afterwards washed and kept submerged in PBS for further analysis. Fluorescence images were obtained using bright field or DAPI/GFP filters with 4× and 10× objectives on Gen5 Image software, using the Biotek Cytation 3.

### 2.8. mRNA Transcriptome and Analysis

RNA easy^®^ Plus Kit (74134, Qiagen, Hilden, Germany) was used to extract RNA from CRC-FX-R and CRC treatment-naïve cells according to the manufacturer’s instructions. The RNA quality control was performed using FastQC v.0.11.5. Library preparation was carried out using TruSeqHT Stranded mRNA dual indexing (Illumina) and the sequencing was performed on an Illumina HiSeq 4000 System using 100-bp single-end reads protocol. STAR v.2.5.3a software was used to map the reads to the human genome (UCSC hg38) with an average alignment around 92%. PicardTools v.2.9.0 was used for biological quality control and raw counts were obtained using HTSeq v.0.9.1. The R/Bioconductor package edgeR v.3.24.3 was used for normalization and differential expression analysis. Statistical significance was assessed with a general linear model, negative binomial distribution, and quasi-likelihood F test (add correction test type). Genes were considered differentially expressed with fold change > 2 and *p*-value < 0.05 (with a false discovery rate of 5%) [39].

### 2.9. Statistical Analysis

All data are presented as the mean of multiple (N) independent experiments with corresponding standard deviation (SD) as indicated in the figure legends (N = number of biological replicates; n = intraexperimental replicates). Data analysis was performed using Graphpad Prism^®^ v. 8.0.1, and statistical significance (* *p* < 0.05, ** *p* < 0.01 and *** *p* < 0.001) was obtained using one-way or two-way ANOVA test with post hoc multiple comparison tests as specified in the figure legends.

## 3. Results

### 3.1. Establishment of Acquired Resistance to FOLFOXIRI in Human CRC Cell Lines

CRC patients treated with chemotherapeutic drug combinations lose sensitivity to the treatment in time. To mimic this situation, we established FOLFOXIRI-resistant (-FX-R) human CRC cell lines. This was carried out by chronic treatment of LS174T, HCT116, DLD1 or SW620 cells (Table 1) with FOLFOXIRI (FX) at previously optimized, cell-line-specific doses [21] (Table 2). The drug doses were 2- to 33-fold lower than clinically used doses of FX (CUD). We considered the cells to be resistant once they became significantly insensitive to FX treatment when compared to FX-naïve cells and reached a stable response over time.

**Figure 1:**
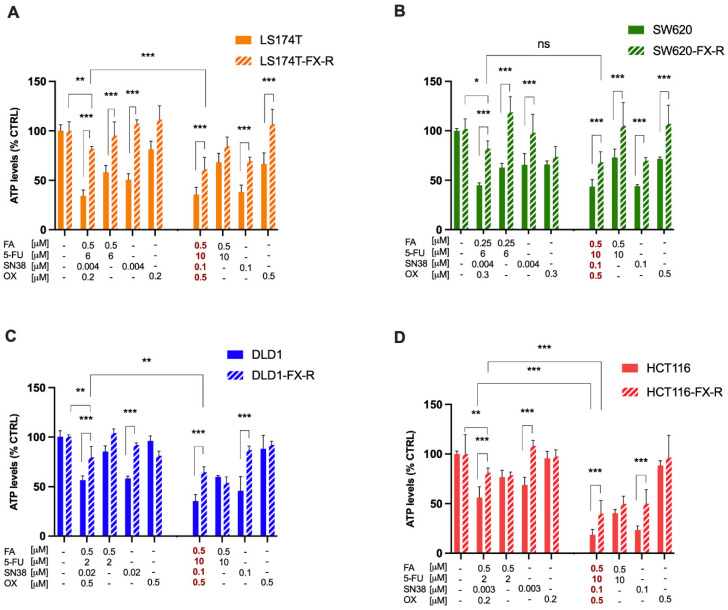
Resistance induction to FX in human CRC cell lines. Activity of cell line-specific optimized FX (Table 2), FX at the clinically used dose (concentration values in red) and monotherapies on cell metabolic activity in LS174T (**A**) SW620 (**B**) DLD1 (**C**) and HCT116 (**D**) cells and their FX-R clones. Error bars represent the SD between the independent experiments (N = 3, n = 3). Significances of * *p* < 0.05, ** *p* < 0.01 and *** *p* < 0.001 represent the comparison between CRC-FX-R and treatment-naïve cells (unpaired *t*-test) or the comparison between the ODC and CUD (two-way ANOVA with post hoc Tukey’s multiple comparisons test).

Every two weeks, a cell metabolic activity assay (CellTiter-Glo^®^) was performed to evaluate the decrease in cell sensitivity to chemotherapy chronic treatment compared to the treatment-naïve cells (Supplementary Figure S2). Significant loss of sensitivity to FOLFOXIRI was obtained after 60, 62, 36 and 34 weeks in LS174T, HCT116, DLD1 and SW620 cells, respectively (Figure 1). The activity of the FX was significantly reduced in all four cell lines (*p* < 0.01) with a respective decrease in inhibition in cell viability of 66% vs. 18% (LS174T, Figure 1A), 58% vs. 18% (SW620, Figure 1B), 44% vs. 20.5% (DLD1, Figure 1C), and 44% vs. 19% (HCT116, Figure 1D). Furthermore, the chronically treated cells were also resistant to FX administered at clinically used doses (CUD), with a respective drop-in activity of 64.3% vs. 38.8% (LS174T), 81.3% vs. 60% (HCT116), 64.5% vs. 35.3% (DLD1) or 56.5% vs. 31.8% (SW620 cells), Figure 1A–D. Each individual drug composing FX was also tested in FOLFOXIRI-naïve (FX) or FOLFOXIRI-resistant (FX-R) cells. Drug dose–response curves were relatively well superimposing in both cell types for FA, 5-FU and SN38 (Supplementary Figure S3). However, dose–response curves for SN38 show significant loss of sensitivity at higher doses in all FX-treated cells.

### 3.2. Morphological Features in CRC Cells upon FOLFOXIRI Resistance Induction

**Figure 2:**
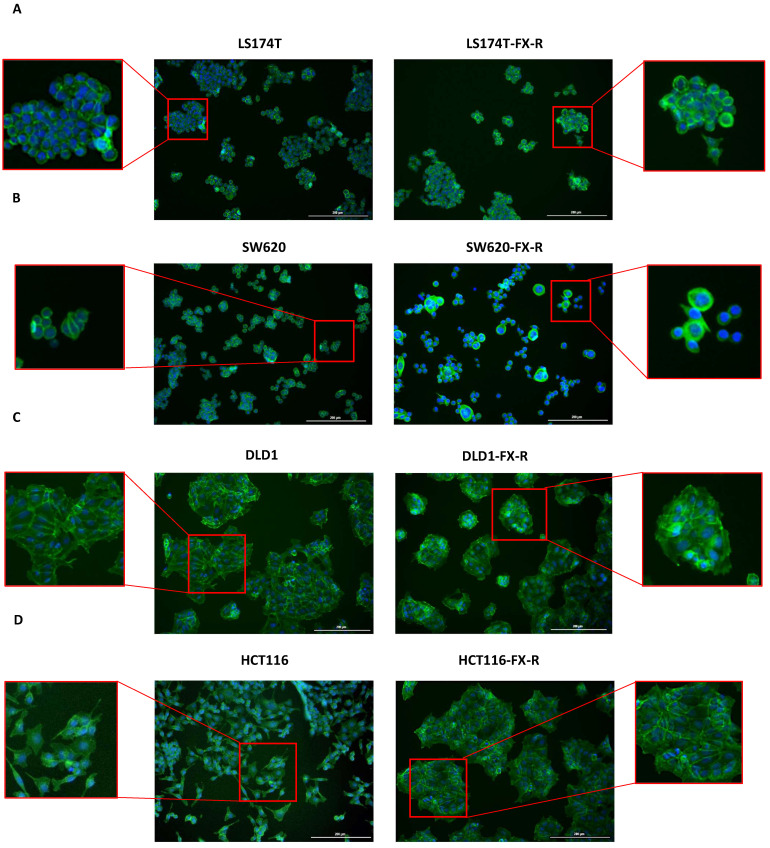
Morphology of FX-resistant and FX-naïve human CRC cells. Representative fluorescence images of FX-naïve and FX-resistant cells: LS174T (**A**) SW620 (**B**) DLD1 (**C**) and HCT116 (**D**) of DAPI (blue) and phalloidin (green) stained cells. n = 5. Scale bar = 200 µm.

**Figure 3:**
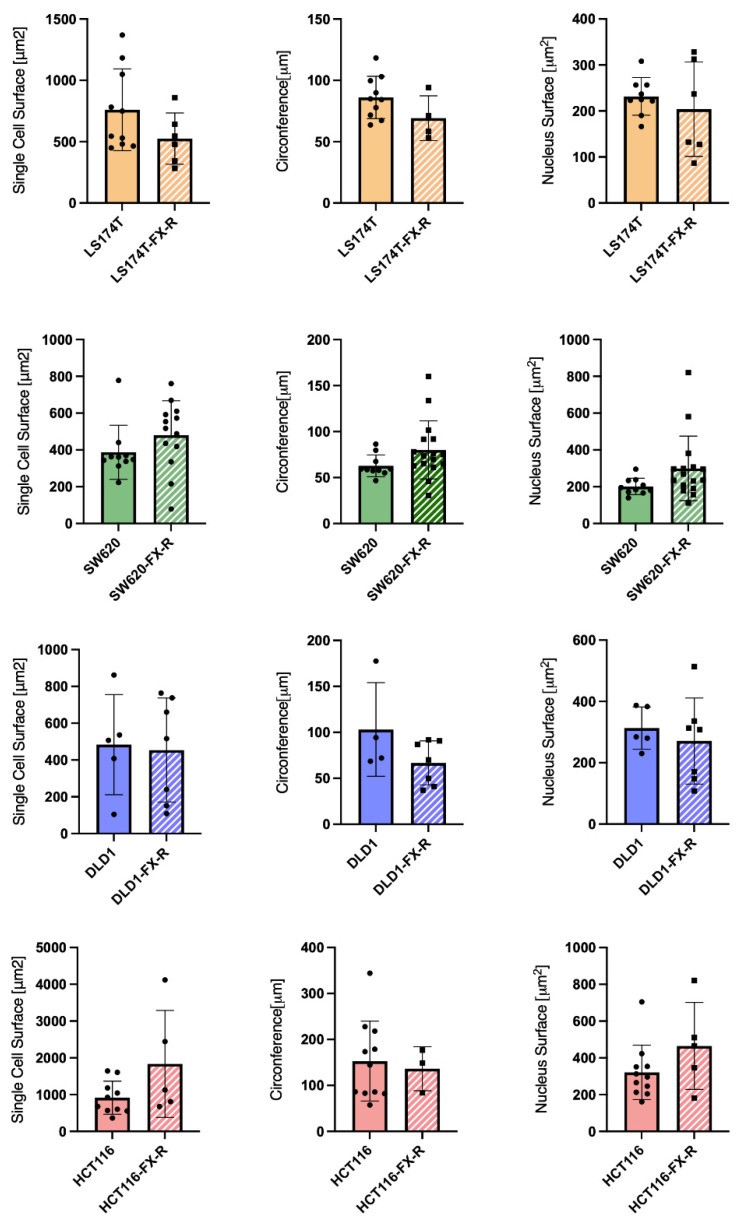
Cell morphology after FOLFOXIRI chronic treatment. Bar graphs of image-based quantification of single cell size (left graphs), circumference (middle graphs) and nucleus size (right graphs) of LS174T (yellow bars) SW620 (green bars) DLD1 (blue bars) and HCT116 (red bars) cells and their FX-R clones (N = 2, n = 5–10). Error bars represent the SD.

To analyze the organization of the actin filament system, CRC cells were seeded on coverslips and cultured for 48 h. After this time, the cells were fixed and stained with phalloidin to visualize filamentous actin (F-actin) and DAPI to visualize the nuclei. LS174T and LS174T-FX-R cells were round, and they grew in small colonies and did not form a border of cortical actin arcs at the cell periphery. The actin cytoskeleton of the LS174T-FX-R cells was more visible and concentrated as a cortical ring under the cellular membrane when compared to treatment-naïve cells (Figure 2A). The overall cell surface of LS174T-FX-R cells was (insignificantly) smaller compared to treatment-naïve cells, whereas the nuclei size did not differ between the two cell types (Figure 3). In addition, LS174T-FX-R developed a more diffusive shape with protrusions (Figure 2A-magnification). SW620 cells in culture may be present in three main morphological categories: spindle-shaped, blebbing and round cells [40]. In our culture conditions (serum-supplemented medium, in non-coated plates), the SW620 cells were mostly round and smaller than LS174T cells. After chronic treatment with FX, the body surface increased compared to SW620 cells and the cells presented different shapes, i.e., round or elongated, see Figure 2B. On the other hand, the nuclei size did not differ between the two cell types (Figure 3), however, several cells in the resistant culture had denser and more concentrated cortical actin (Figure 2B). DLD1 cells formed large cell clusters with actin mostly located at the periphery of the cells, close to the other cell edges, with the definition of the borders of each cell. DLD1-FX-R cells formed smaller, more compact, and circular clusters. Those cells displayed thinner and poorly oriented stress fibers. Treatment-naïve cells were clearly separated with actin fibers, whereas the delimitation of these cells was less visible in the resistant cells. The cell body and nuclei sizes remained the same in DLD1 and DLD1-FX-R cells (Figure 2C). The most striking morphological difference after chronic treatment with FX was observed in HCT116 cells. While treatment-naïve HCT116 cells were elongated and grew as single cells, HCT116-FX-R cells formed clusters and presented a rather round shape with bright actin filament staining, but also a patchy appearance in the cytoplasm (Figure 2D).

### 3.3. Alterations in Gene Expression and Biological Function upon FX Resistance Induction

To characterize global transcriptome and molecular changes in CRC cells upon chronic FX treatment, we performed bulk RNA sequencing comparing CRC-treatment-naïve and FX-resistant cells. For that, we selected LS174T and SW620 cells and isolated RNA in three replicates.

**Figure 4:**
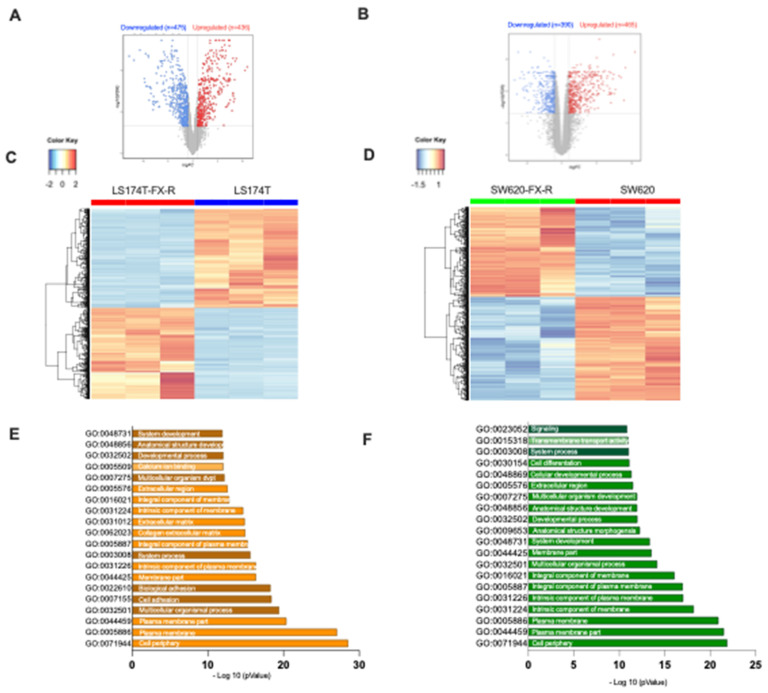
RNA-sequencing data for LS174T and SW620 cells and their FX-R clones. RNA-sequencing (RNA-seq) was performed to study the differential gene expression based on analysis of CRC FX-treated and FX-naïve cells. Volcano plots of significant genes (*p*-value with FDR < 0.05) and a fold change > 2 (logFC) in (**A**). LS174T-FX-R vs. LS174T and (**B**). SW620-FX-R vs. SW620 cells. Each dot is a gene with the most upregulated genes are towards the right, the most downregulated genes are towards the left, and the most statistically significant genes are towards the top. Here, the blue dots represent the downregulated genes and the red ones represent the upregulated genes. Vertical lines highlight log2 fold changes of −1 and +1 (FC = 2 <=> log(FC) = 1), while a horizontal line represents a corrected *p*-value of 0.05. For LS174T cells, panel A = 911 genes–downregulated (n = 475) and upregulated (n = 436); for SW620, panel B= 855- downregulated (n = 390) and upregulated (n = 465). Heatmap of genes in (**C**). LS174T-FX-R vs. LS174T and (**D**). SW620-FX-R vs. SW620 describe cells differentially up- and downregulated. The color and intensity of the boxes are used to represent expression values: blue for a gene with a small expression value and red with a high value (according to the color key) (**E**,**F**). Enrichment analysis of both up and downregulated genes in LS174T (N = 3, n = 3) and SW620 (N = 3, n = 3) for Gene Ontology. The top 20 functional clusters are sorted according to *p*-value and color coding corresponds to cellular components, biological processes, and molecular functions.

RNA sequencing analysis of LS174T cells demonstrated differential gene expression, see heatmap Figure 4C. These alterations included a cluster of 907 genes (*p*-value with false discovery rate (FDR) < 0.05 and FC ≥ 2), of which 52% of genes were downregulated and 48% upregulated in LS174T-FX-R cells (Figure 4A). For SW620 cells, 855 genes were differentially expressed, including 390 downregulated and 465 upregulated (Figure 4B,D). Enrichment analysis for Gene Ontology (in three groups, i.e., biological process; cellular component and molecular function) is depicted on top of the enriched GO terms; see Figure 4E,F. These were mainly responsible for cell periphery, plasma membrane, membrane components, developmental process, or anatomical structure development.

RNA-sequencing of SW620 cells highlighted one gene whose expression was significantly different between the resistant and FX-naïve clones versus five genes for the LS174T cells. This does not indicate that the difference between the SW620 clones is lower, but that many genes are different, albeit expressed at low levels. Additionally, this is normally expected due to the variety of experimental conditions and genetic perturbations that the cells might have gone through [41]. The most upregulated gene in SW620-FX-R, compared to the treatment-naïve cells, was the solute carrier family 2 member gene (SLC2A), see Supplementary Table S1. That is responsible for the expression of the glucose transporter family (GLUT) that controls the uptake of sugar at the cell membrane level [42]. GLUT1–4 are primarily responsible for transporting glucose into the intracellular compartment, whereas GLUT5 is exclusively in charge of fructose uptake and is linked to a high cancer risk [43]. The most significantly downregulated gene in LS174T-Fx-R cells was protein tyrosine phosphatase receptor S (PTPRS), a tumor suppressor gene known to mediate cell migration and invasion by downregulation of epithelial-mesenchymal transition [44]. Our results also revealed significant downregulation of oxoglutarate dehydrogenase-like gene (OGDHL) in LS174T-FX-R, which is implicated in the tricarboxylic acid (TCA) cycle, and indirectly, the induction of apoptosis [45,46]. The full RNAseq dataset is available at: https://zenodo.org/record/7111566#.YzB5zsFBwq0, accessed on 25 September 2022.

### 3.4. Optimized Combination of Tyrosine Kinase Inhibitors Overcomes FX-Resistance in CRC Cells

**Figure 5:**
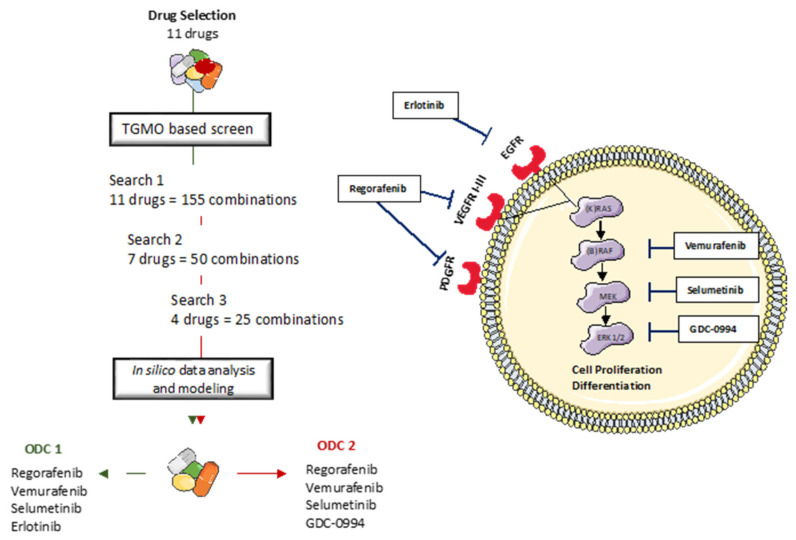
The TGMO-based screen. Through consecutive rounds of screening (Search 1–3) and data modeling [47], the strongest and most robust drug interactions define the final drug combination selection, to converge into two final ODC_1_ and ODC_2_.

**Figure 6:**
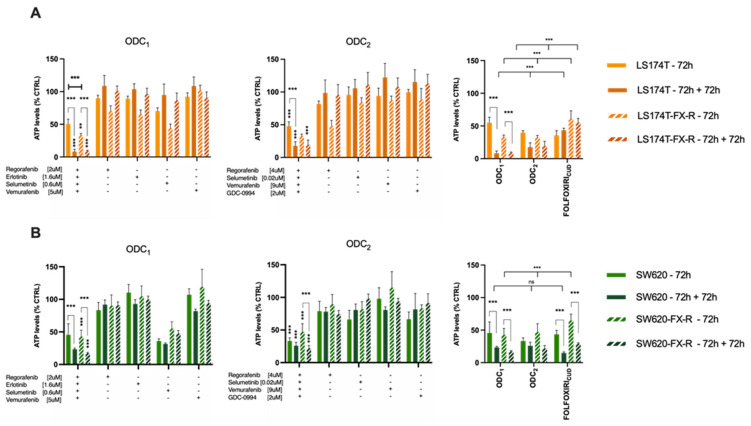
Activity of the ODCs in FX-naïve and FX-resistant cells in 2D culture of LS174T (**A**) or SW620 (**B**) cells (N = 3, n = 3). Error bars represent the SD and significances of ** *p* < 0.01, *** *p* < 0.001 (two-way ANOVA with post hoc Tukey’s and Sidak’s multiple comparisons test).

We next evaluated the sensitivity of FX-resistant CRC cells to targeted treatments. We have previously identified optimized multidrug combinations that were selective and active in CRC cell lines [32], see Figure 5, Supplementary Information and Supplementary Figure S1. In this study, we validated the activity of two previously optimized drug combinations. DLD1 cell-specific optimized drug combination (ODC_1_) consisting of regorafenib (2 μM), erlotinib (1.6 μM), selumetinib (0.6 μM) and vemurafenib (5 μM); and SW620-specific ODC (ODC_2_), consisting of regorafenib (4 μM), selumetinib (0.02 μM), vemurafenib (9 μM) and GDC-0994 (2 μM) each contributed to the overall activity. ODC_1_, although optimized in DLD1 cells, was active in SW620 and SW620-FX-R cells, especially after retreatment, where the cell metabolic activity was reduced from 42% to 18% in SW620-FX-R cells (Figure 6A, left graph). ODC_2_ inhibited the metabolic activity in 2D culture by over 60% (SW620 cells) or 50% (SW620-FX-R cells), and retreatment for another 72h potentiated this effect by 12% and 27%, in SW620 and SW620-FX-R cells, respectively, Figure 6A, middle graph. The activity of both ODC_1_ and ODC_2_ after treatment was comparable to FOLFOXIRI administered at CUD (Figure 6A, right graph). Interestingly, ODC_1_ and ODC_2_ were very potent with similar activity in LS174T and LS174T-FX-R cells, especially after retreatment, with a reduction of over 30% in cell metabolic activity for ODC_1_ in both clones (Figure 6B, left graph). The activity of ODC_1_ and ODC_2_ was significantly higher compared to the FOLFOXIRI at CUD when LS174T-FX-R cells were retreated, with 45% and 35% increase in activity, respectively (Figure 6B, right graph). 

**Figure 7:**
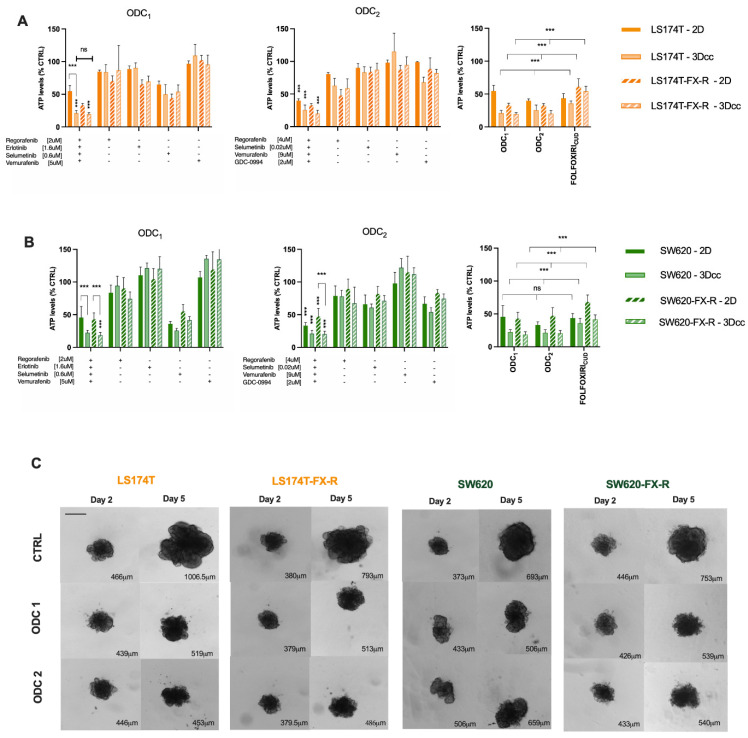
Activity of ODCs in 2D and 3D co-cultures of LS174T (**A**) and SW620 (**B**) FX-naïve and FX-R cells. Error bars represent the SD and significances of *** *p* < 0.001 (two-way ANOVA with post hoc Tukey’s and Sidak’s multiple comparisons test) N = 3, n = 3. (**C**) Representative bright field images of 3D co-cultures on day 2 and day 5 of experiment in control-(CTRL-) and ODC-treated conditions. Scale bar represents 400 μm. Quantification analysis was performed using imageJ (0.0783 pixels/μm). The numbers in the bottom right corner correspond to average diameter of each spheroid.

We have previously established heterotypic 3D co-cultures composed of CRC cells and fibroblasts (1:1) and 5% endothelial cells, Figure 7A. Those cell ratios in co-cultures were defined based on the histological tumor composition of patient CRC tumor samples according to the staging. Both ODC_1_ and ODC_2_ maintained their activity in FX-naïve and FX-resistant cells SW620 (A) and LS174T (B) cells, Figure 7B,C. The activity of both ODC increased in the 3D co-cultures models when compared to the 2D models. ODC_1_ and ODC_2_ overcame the resistance to FX by inhibiting the cell metabolic activity in SW620 and LS174T 3Dcc, respectively, by up to 82% vs. 60% and 81 vs. 45% compared to the activity of FX at CUD. In addition, even though size is not a determining factor of the activity, an inhibition of the growth is evident, where the treated 3Dcc of both cell lines in treatment-resistant and -naïve clones, with ODC_1_ and ODC_2_, were smaller after 72h incubation compared to the control.

## 4. Discussion

In this study, we created CRC in vitro models that mimic the treatment path of late-stage CRC tumors. To do so, we induced FOLFOXIRI (consisting of 5-FU, folinic acid, SN38 and oxaliplatin [21]) resistance in four metastatic and non-metastatic human CRC cell lines characterized by different mutational statuses.

Multiple resistance induction strategies were previously reported in the literature [48], including one-time treatment with a high dose, or pulsed treatment strategy with a fixed dose, or chronic treatment with an increasing dose of the drug [49]. Among the different modalities used, we have chosen to apply a clinically relevant strategy that mimics the chemotherapy cycles that a cancer patient undergoes, by applying a pulsed chronic treatment strategy with a ”recovery” period till the next treatment point. To do so, we exposed the different CRC cell lines once weekly to their corresponding FOLFOXIRI mixture, Table 1. The treatment was kept for 72 h then the cancer cells recovered in “treatment-free” medium till the next treatment cycle. Resistance to FOLFOXIRI was established upon at least 36 treatment cycles, Supplementary Figure S2. In their meta-analysis, McDermott et al. highlighted resistance to a drug when the chronically treated cell line showed a two- to eight-fold lower response to the given treatment compared to the parental cell line, they defined it as “clinically relevant” resistance to chemotherapy [48]. In this study, we induced resistance for the first time to FOLFOXIRI in CRC cell lines, and in both LS174T and SW620 we were able to increase resistance to the quadro-therapy at the clinically used dose by up to 1.7-fold. Multiple studies have reported the induction of resistance to up to three drugs consisting FOLFOXIRI in CRC cell lines. Yu et al. reported the induction of resistance to FOLFOX (folinic acid, 5-FU and oxaliplatin) in HCT116 and HT29 CRC cell lines [50,51]. Their strategy consisted of a 12-week resistance induction with increasing doses of 5-FU (25–50–100 μM) and oxaliplatin (0.625–1.25–2.5 μM). We report the induction of resistance to 5-FU, folinic acid, SN38 and oxaliplatin and defined resistance to the chemotherapy when the response was significantly different between the -naïve and the chronically treated cells to FOLFOXIRI at clinically used dose (CUD).

Pasqualato et al. reported a quantitative analysis of the morphological changes in chemotherapy-resistant CRC cells. The cell shape analysis was performed using Normalized Bending Energy (NBE) parameter that links cell morphology to thermodynamics changes. A progressive increase in NBE was correlated with resistance to increasing doses of 5-FU (0.1–2 μM) [52]. Chemo-resistant cells were described to have a more “diffusive” shape associated with high NBE values, this is in line with our findings, where after chronic treatment with FOLFOXIRI, both LS174T and SW620 cells became less round, and more cells exhibited, respectively, more protrusions or a spindle shape, acquiring a more invasive morphotype (Figure 2A,B).

We further investigated the influence of the resistance induction in LS174T-FX-R and SW620-FX-R cells on the RNA transcriptome (Figure 4 and Supplementary Table S1). Differentially expressed genes were similar in both cell lines and were related to the cell periphery, plasma membrane, membrane components, developmental process, or anatomical structure development. Our results show an important upregulation of SLC2A in SW60-FX-R cells, responsible for the expression of GLUT5. The exact role of GLUT5 in colon cancer is not yet fully elucidated, but other studies have shown that the inhibition of GLUT5 in CRC patients is linked to a decrease in the viability of the cancer cells. Park et al. have shown that the expression of the glucose receptor led to the activation of AKT1 and AKT3, underlining the role of GLUT5 as a biomarker for drug resistance development in CRC after chemotherapy [53]. In fact, the activation of the AKT1 pathway has been widely reported in CRC as an early occurring event in carcinogenesis. Narayan et al. have shown in their study that increased levels of phosphorylated AKT1/mTOR/4EBP1 along with p21 have been observed in FOLFOX-resistant CRC cells. Furthermore, several therapeutic agents inhibiting AKT1, such as perifosine and MK-2206 currently in phase III and phase II clinical studies, respectively, show improved prognosis in cancer patients through sensitizing cancer cells to different treatment modalities [54]. Furthermore, studies have shown that RAS mutant cells have increased expression of GLUT1 as a survival modality, which comes at the expense of drug resistance, through constitutive activation of the RAS-MAPK pathway as a secondary resistance mechanism. This converges with our results, where our optimized ODC treatment overcomes resistance to FOLFOXIRI in both RAS mutant cell lines, as we have previously shown that it specifically targets the MAPK pathway [14]. The most significantly downregulated gene in LS174T-FX-R cells was protein tyrosine phosphatase receptor S (PTPRS). Studies have shown that CRC cells with downregulated levels of PTPRS show a higher response to MEK/ERK inhibition due to a lack of adaptive resistance response that allows a bypass of MEK/ERK drug blockade, unlike the parental cells. This has been elucidated in HCT116 cells using multiple genetic modifications, showing the possible role of SRC in therapeutic resistance to MEK/ERK inhibitors [55]. Metabolic reprogramming has been shown to be a hallmark of cancer, through the upregulation of genes implicated in tumor progression through fatty acid, one-carbon and glucose synthesis. OGDHL have been shown to be a tumor suppressor gene. The downregulation of the latter has been shown to be implicated in the progression of several cancers such as liver, pancreatic, cervical, hepatocellular, and colorectal cancer [56,57,58]. Using quantitative real-time PCR, Fedorova et al. reported the relative levels of OGDHL mRNA in 30 CRC samples and highlighted a decrease of up to 170 folds in more than 50% of the samples. Their hypothesis was since the TCA cycle and oxidative phosphorylation (OXPHOS) are inhibited while glycolysis is activated as the tumor grows and oxygen deficit increases. However, this contradicts the findings of Nietzel et al., where comparative analysis performed directly on CRC patient-derived material has revealed an upregulation of OXPHOS in CRC cells, especially in microsatellite-stable (MSS) samples. This upregulation was further associated with chemoresistance in patients treated with oxaliplatin and 5-FU [45]. This was also in line with the findings of Denise et al., where pharmacological inhibition of OXPHOS in combination with 5-FU was shown to overcome chemoresistance by inhibiting the viability of 5-FU resistant CRC cells [59].

Although the mechanistic explanation of the metabolic shift in CRC-FX-treated cells was not fully elucidated, we hypothesize that it could be linked to possible adaptive metabolic reprogramming in the cells. Further in-depth investigations will reveal the mechanism of activity of ODCs that overcomes metabolic destabilization in FX-R CRC cells.

## 5. Conclusions

Chemoresistance was obtained over time by chronically exposing human CRC cells to a FOLFOXIRI mixture. RNAseq data gave insight for a better understanding of some potential signaling pathways implicated in the long-term treatment with chemotherapy. This study is meant to serve as a platform to study how an induced FOLFOXIRI-drug resistance could alter the response to targeted treatments.

## Figures and Tables

**Table 1:** CRC cell lines used in the study and their characterization [26,27,28,29,30].

Cell Line	Patient	Cancer Stage/Type	Genomic (In)stability	Mutations/Deregulations	FOLFOXIRI Exposure [Weeks]
**LS174T**	Female	2 (primary)	MSI	KRAS, PIK3CA, BRAF	60
**SW620**	Male	3 (metastatic)	MSS; CIN	APC, KRAS, TP53	34
**DLD1**	Male	3 (metastatic)	MSI, CIMP	APC, KRAS, PIK3CA, TP53	36
**HCT116**	Male	1 (primary)	MSI, CIMP	KRAS, PIK3CA	62

MSI: microsatellite instability; MSS: microsatellite stability; CIN: chromosomal instability, CIMP: CpG island methylator phenotype.

**Table 2:** FX doses used in this study.

Drug [µM]	CUD	FX_DLD1_	FX_HCT116_	FX_SW620_	FX_LS174T_
**FA**	0.5	0.5	0.5	0.25	0.5
**5-FU**	10	2	2	6	6
**SN38**	0.1	0.02	0.003	0.004	0.004
**OX**	0.6	0.5	0.2	0.3	0.2
**Efficacy** **[% CTRL] ± SD**		48 4.9	35 0.7	37 8.6	45 2.2

CUD: clinically used dose (see Materials and Methods); ODC: optimized drug combination; FA: Folinic acid, 5-FU: 5-Fluorouracil, SN38: active metabolite of Irinotecan, OX: Oxaliplatin.

## Data Availability

The data can be shared up on request.

## Supplementary Materials

The following supporting information can be downloaded at: https://www.mdpi.com/article/10.3390/cancers14194812/s1, Information S1: The Therapeutically Guided Multidrug Optimization (TGMO) method; Figure S1: FOLFOXIRI resistance induction in CRC cell lines; Figure S2: Drug-Response curves in FX-R vs. naïve CRC cell lines; Table S1: Top 10 up- and downregulated gene expression in SW620 and LS174T and their FX-R clones. Full RNAseq dataset is available at: https://zenodo.org/record/7111566#.YzB5zsFBwq0, accessed on 25 September 2022, https://doi:10.5281/zenodo.7111566, accessed on 25 September 2022. References [32,38,47] are cited in Supplementary Materials.
