# Supplementary material for: FOLFOXIRI Resistance Induction and Characterization in Human Colorectal Cancer Cells
Source: Cancers (Basel). 2022 Sep 30;14(19):4812. doi: 10.3390/cancers14194812 (PMC9564076; doi:10.3390/cancers14194812)
Supplement: Supplementary file 1 [file cancers-14-04812-s001.zip › cancers-1936952-supplementary.pdf]

Supplementary information

FOLFOXIRI Resistance Induction and Characterization in Human Colorectal Cancer Cells

George M. Ramzy [1,2,3], Laura Boschung [1,2], Thibaud Koessler [3,4], Céline Delucinge-Vivier [5], Mylène Docquier [5,6], Thomas A. McKee [7], Laura Rubbia-Brandt [7] and Patrycja Nowak-Sliwinska [1,2,3,*]

[1] Molecular Pharmacology Group, School of Pharmaceutical Sciences, Institute of Pharmaceutical Sciences of Western Switzerland, University of Geneva, 1211 Geneva, Switzerland
[2] Institute of Pharmaceutical Sciences of Western Switzerland, University of Geneva, 1211 Geneva, Switzerland
[3] Translational Research Center in Oncohaematology, 1211 Geneva, Switzerland
[4] Department of Oncology, Geneva University Hospitals, 1205 Geneva, Switzerland
[5] iGE3 Genomics Platform, University of Geneva, 1211 Geneva, Switzerland
[6] Department of Genetics & Evolution, University of Geneva, 1211 Geneva, Switzerland
[7] Division of Clinical Pathology, Diagnostic Department, University Hospitals of Geneva (HUG), 1211 Geneva, Switzerland
* Correspondence: patrycja.nowak-sliwinska@unige.ch; Tel.: +41-22-379-3352

Supplementary Information S1
Supplementary Figures S1-S3
Supplementary Table S1
Supplementary References

Supplementary information

Supplementary information S1

The Therapeutically Guided Multidrug Optimization (TGMO) method[1, 2], see **Supplementary Figure S1,** was used to establish drug-drug interactions between a set of 11 different targeted drugs.

The different drug combinations were tested according to an orthogonal array composite design matrix (OACD), specifically designed for the most optimal information acquisition from an infinite number of experimental possibilities. The first part of the matrix exposes the linear effect of single and two-drugs over a large search space, whereas the second part characterizes the non-linear response over multiple doses of the drugs. The resulting OACD matrix is a resolution IV matrix[3] ideal to define the most influential variables tested, while providing a cross-validation between both parts of the matrix design. The screening is performed in 3 sequential rounds of searches, each round allowing the selection of the most active and interesting drug-drug interactions, while eliminating the most inactive and antagonistic drugs from the drug pool. The most robust interactions appear in multiple rounds including the final model in Search 3.

Experimentally, drug-dose response curves for all 11 drugs were performed to define the range of activity of the drugs on the different cell lines considered, and determine the input doses to be used, and which correspond to the IC_{20} and half of the previous. The drug input is low to only identify the strongest of drug-interactions. In Search 1 we tested 11 drugs in 155 different drug combinations on both CRC and non-malignant colon CCD841CoN cells simultaneously. The difference between the activity of both is named therapeutic window (non-malignant cell viability - cancer cell viability) and is used to evaluate the selectivity of the drug combination activity. The output is measured by cell metabolic activity (ATP level, % CTRL). The experimental data was then analyzed using stepwise second-order linear regression analysis by Matlab®. This algorithm generates regression coefficients describing the contribution of each drug individually (single drug first-order term), all two-drug interactions, and finally how the effect of a drug varies over different dose levels (single drug second-order terms). The generated models guide drug selection and elimination, as the negative regression coefficients are considered as synergistic activity, and positive regression coefficients signify antagonistic activity. Through consecutive rounds of screening (Search 1-3) the most strong and robust drug interactions define the final drug combination selection.

Supplementary Figures

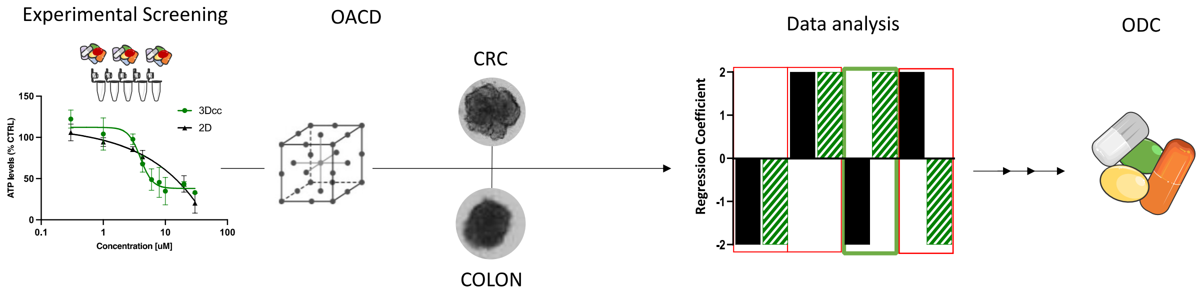

Supplementary Figure S1: The Therapeutically Guided Multidrug Optimization (TGMO) method.

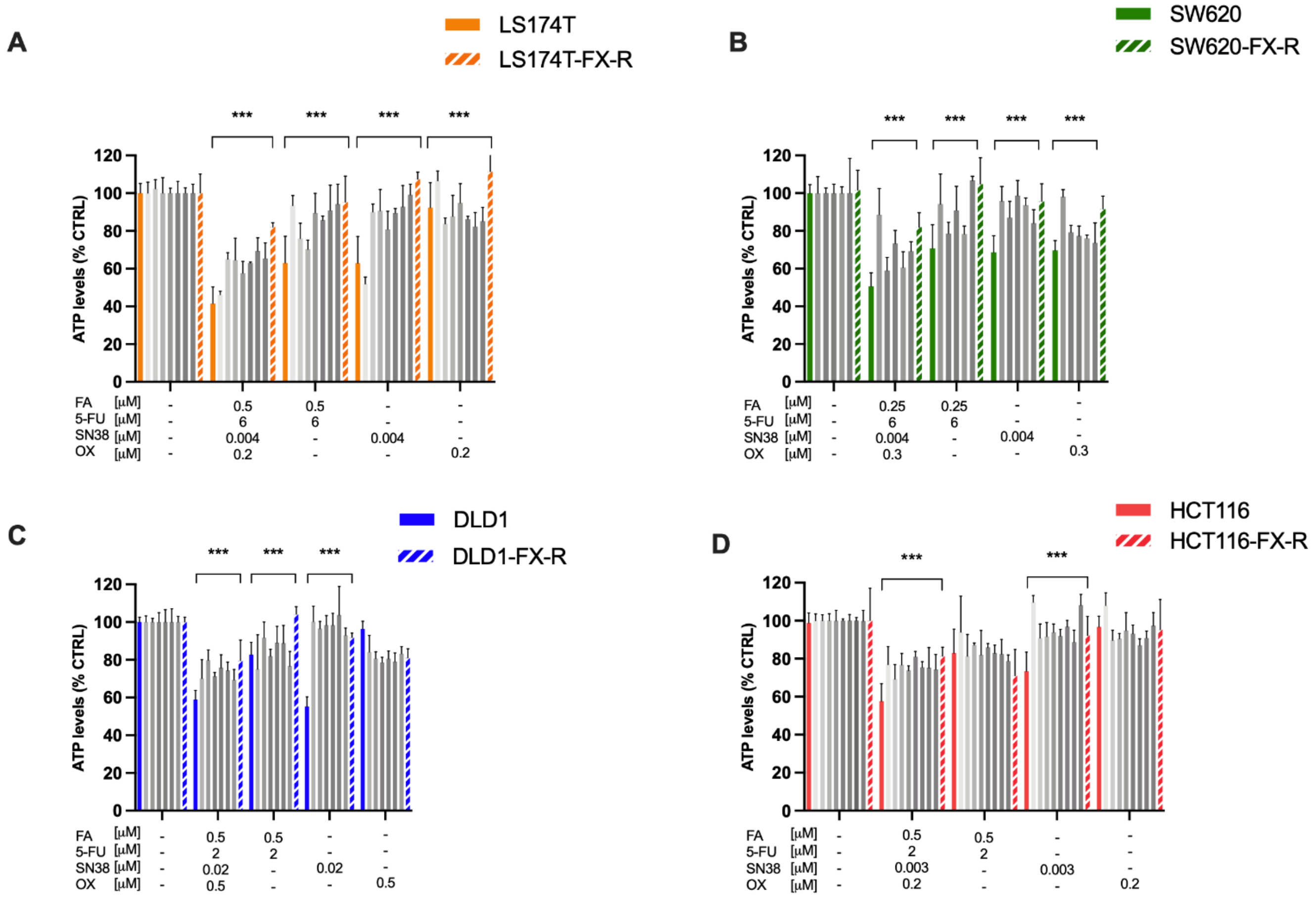

Supplementary Figure S2: Resistance induction to FX in human CRC cell lines.

Activity of cell line-specific optimized FX and monotherapies on cell metabolic activity in LS174T (**A**) SW620 (**B**) DLD1(**C**) and HCT116 (D) cells and their FX-R clones. Cells were treated once weekly with their corresponding FOLFOXIRI mixture. Treatment was kept for 72h. Every two weeks, a cell metabolic activity assay was performed to evaluate the decrease in cell sensitivity to the chemotherapy chronic treatment. Error bars represent the SD and significances of ***$p < 0.001$ represent the comparison between treatment-naïve and resistant cells (two-way ANOVA with post-hoc Tukey's multiple comparisons test). N=3

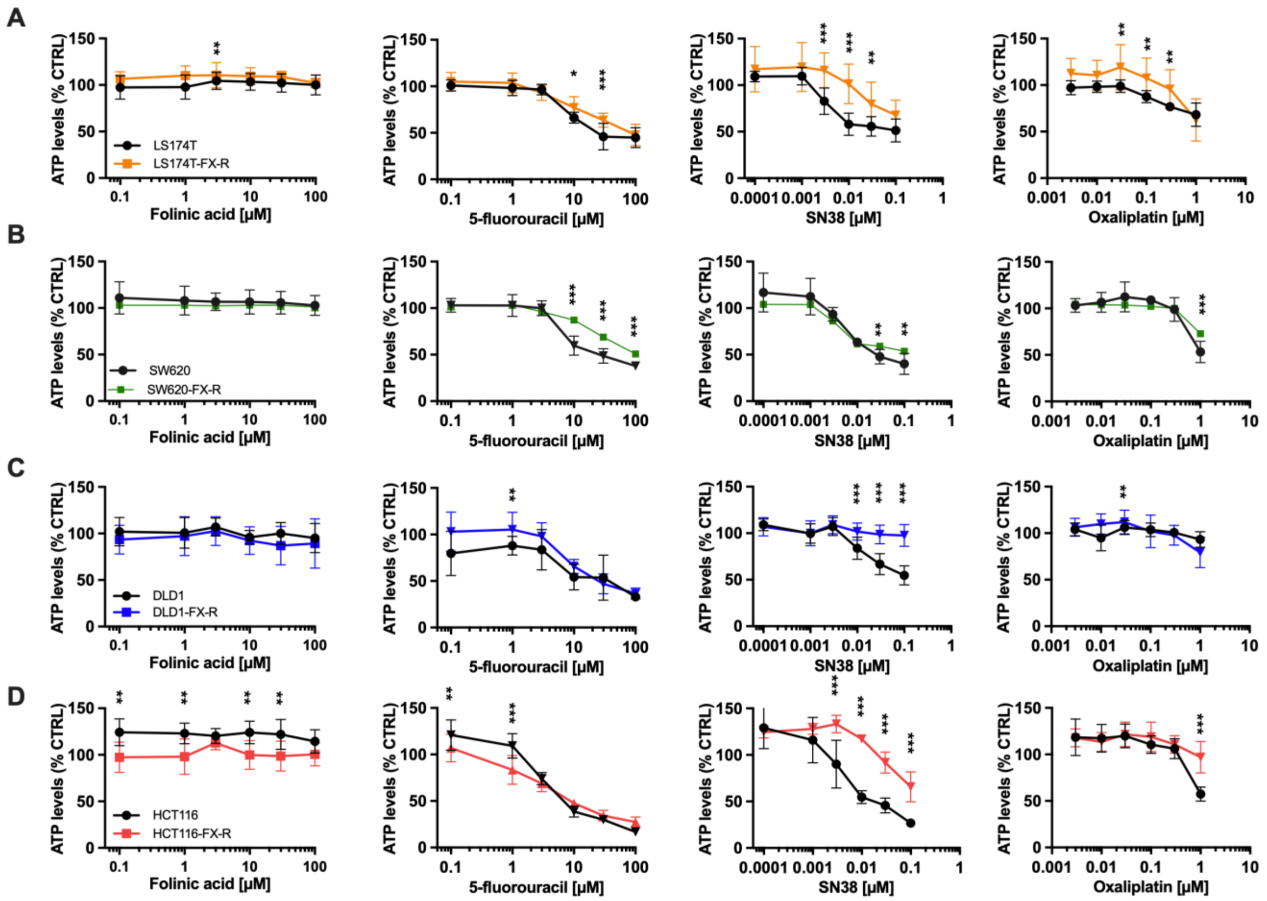

Supplementary Figure S3: Drug-Response Curves
Drug dose response curves of folinic acid (FA), 5-fluorouracil (5-FU), SN38 and oxaliplatin (OX) in LS174T **(A)** SW620 **(B)** DLD1 **(C)** HCT116 **(D)** cells and their corresponding FX-R clones. Drug concentrations are presented on a logarithmic scale. Error bars represent the SD and significances of *$p < 0.05$, **$p < 0.01$ and ***$p < 0.001$ represent the comparison between naïve and FX-R cells (unpaired t-test, N=3).

Supplementary Tables

Supplementary Table S1: Top 10 up- and down- regulated gene expression in SW620 and LS174T and their resistant clones.

Gene	Description	SW620-FX-R vs. SW620
SLC2A5	solute carrier family 2 member 5	1'401.32
CACNA2D1	calcium voltage-gated channel auxiliary subunit alpha2delta 1	552.67
MECOM	MDS1 and EVI1 complex locus	474.12
RNF217	ring finger protein 217	350.63
ADAMTS19	ADAM metallopeptidase with thrombospondin type 1 motif 19	295.92
CELP	carboxyl ester lipase pseudogene	205.56
KCNV1	potassium voltage-gated channel modifier subfamily V member 1	180.87
AQP5	aquaporin 5	171.91
BMPR1B	bone morphogenetic protein receptor type 1B	166.24

DOCK3	dedicator of cytokinesis 3	157.57
LIPC	lipase C, hepatic type	-40.69
CDIP1	cell death inducing p53 target 1	-41.78
LOC202181	SUMO interacting motifs containing 1 pseudogene	-42.65
SLC9A3	solute carrier family 9 member A3	-43.78
GPR143	G protein-coupled receptor 143	-46.81
CALB1	calbindin 1	-51.52
CRABP1	cellular retinoic acid binding protein 1	-61.57
MYH14	myosin heavy chain 14	-74.87
RDH12	retinol dehydrogenase 12	-84.94
NRCAM	neuronal cell adhesion molecule	-106.20

Gene	Description	LS174T-FX-R vs. LS174T
LRFN5	leucine rich repeat and fibronectin type III domain containing 5	1'982.04
ADGRL2	adhesion G protein-coupled receptor L2	1'471.03
SORCS1	sortilin related VPS10 domain containing receptor 1	435.36
PCDH11X	protocadherin 11 X-linked	373.79
SEMA3D	semaphorin 3D	340.94
LOC101927549	NA	337.66
RMST	rhabdomyosarcoma 2 associated transcript	299.49
LRRC4C	leucine rich repeat containing 4C	287.75
SOX2	SRY-box 2	279.16
MIR100HG	mir-100-let-7a-2-mir-125b-1 cluster host gene	273.63
ZNF22	zinc finger protein 22	-490.03
ASMTL	acetylserotonin O-methyltransferase like	-514.51
THBS1	thrombospondin 1	-560.54
TRIM59	tripartite motif containing 59	-678.14
FAM127A	NA	-728.59
OGFRL1	opioid growth factor receptor like 1	-812.65
ERICH3	glutamate rich 3	-865.15
SLC19A1	solute carrier family 19 member 1	-1'639.15
OGDHL	oxoglutarate dehydrogenase like	-3'683.05
PTPRS	protein tyrosine phosphatase receptor type S	-7'305.45

Supplementary References

1. Weiss, A.; Le Roux-Bourdieu, M.; Zoetemelk, M.; Ramzy, G. M.; Rausch, M.; Harry, D.; Miljkovic-Licina, M.; Falamaki, K.; Wehrle-Haller, B.; Meraldi, P., et al., Identification of a Synergistic Multi-Drug Combination Active in Cancer Cells via the Prevention of Spindle Pole Clustering. Cancers (Basel) 2019, 11 (10).
2. Zoetemelk, M.; Ramzy, G. M.; Rausch, M.; Koessler, T.; van Beijnum, J. R.; Weiss, A.; Mieville, V.; Piersma, S. R.; de Haas, R. R.; Delucinge-Vivier, C., et al., Optimized low-dose combinatorial drug treatment boosts selectivity and efficacy of colorectal carcinoma treatment. Mol Oncol 2020, 14 (11), 2894-2919.

3. Xu, H.; Jaynes, J.; Ding, X., COMBINING TWO-LEVEL AND THREE-LEVEL ORTHOGONAL ARRAYS FOR FACTOR SCREENING AND RESPONSE SURFACE EXPLORATION. Statistica Sinica 2014, 24 (1), 269-289.
